# Development of a Computational Model to Predict Excess Body Fat in Adolescents through Low Cost Variables

**DOI:** 10.3390/ijerph16162962

**Published:** 2019-08-17

**Authors:** Carlos Magno Sousa, Ewaldo Santana, Marcus Vinicius Lopes, Guilherme Lima, Luana Azoubel, Érika Carneiro, Allan Kardec Barros, Nilviane Pires

**Affiliations:** 1 Department of Electrical Engineering, Biological Information Processing Lab, Federal University of Maranhão, São Luis 65085680, MA, Brazil; 2 Laboratory of Signals Acquisition and Processing, LAPS, State University of Maranhão, Campus Paulo VI, São Luís 65700000, MA, Brazil; 3 Centro de Prevenção de Doenças Renais, University Hospital of Maranhão, São Luís 65080805, MA, Brazil

**Keywords:** obesity, adolescent, screening

## Abstract

Background: Excess body fat has been growing alarmingly among adolescents, especially in low income and middle income countries where access to health services is scarce. Currently, the main method for assessing overweight in adolescents is the body mass index, but its use is criticized for its low sensitivity and high specificity, which may lead to a late diagnosis of comorbidities associated with excess body fat, such as cardiovascular diseases. Thus, the aim of this study was to develop a computational model using linear regression to predict obesity in adolescents and compare it with commonly used anthropometric methods. To improve the performance of our model, we estimated the percentage of fat and then classified the nutritional status of these adolescents. Methods: The model was developed using easily measurable socio-demographic and clinical variables from a database of 772 adolescents of both genders, aged 10–19 years. The predictive performance was evaluated by the following metrics: accuracy, sensitivity, specificity, and area under ROC curve. The performance of the method was compared to the anthropometric parameters: body mass index and waist-to-height ratio. Results: Our model showed a high correlation (R = 0.80) with the body fat percentage value obtained through bioimpedance. In addition, regarding discrimination, our model obtained better results compared to BMI and WHtR: AUROC = 0.80, 0.64, and 0.55, respectively. It also presented a high sensitivity of 92% and low false negative rate (6%), while BMI and WHtR showed low sensitivity (27% and 9.9%) and a high false negative rate (65% and 53%), respectively. Conclusions: The computational model of this study obtained a better performance in the evaluation of excess body fat in adolescents, compared to the usual anthropometric indicators presenting itself as a low cost alternative for screening obesity in adolescents living in Brazilian regions where financial resources are scarce.

## 1. Introduction

The prevalence of excess body fat has been growing alarmingly worldwide, this increase is also observed among teenagers [1,2]. In certain developed, the prevalence of obesity among adolescents has achieved high levels [3,4], as in the USA, that 17 % of teenagers are considered obese [4]. Statistics are also alarming in developing countries like Brazil, since the rate of obesity also grows rapidly, that nearly 20% of teenagers are obese [1]. Consequently, the assessment of nutritional status plays a relevant role in the fluctuations in the body composition of the individual, as well as in the survival rate under pathological conditions, since it allows the early diagnosis of comorbidities associated with overweight such as cardiovascular disease and endocrine disorders [5,6].

There are several techniques for assessing body composition, among which bioimpedance (BIA) stands out, which is a fast, portable, and non-invasive method that uses the principle of electrical impedance. Moreover, it has little technical difficulty and high correlation with dual energy X-ray absorptiometry (DXA), and its use is indicated in epidemiological studies and clinical practice [7,8,9,10,11]. However, the use of anthropometric parameters is still more viable due to their low cost when compared to BIA. 

The most commonly used anthropometric indicator in the assessment of adolescent nutritional status is the body mass index (BMI) due to its low cost, simplicity, and high reproducibility. However, BMI has controversies regarding its efficiency, since it has low sensitivity in predicting excess body fat [12,13,14,15,16]. Individuals who have a high percentage of lean mass will have their total weight affected, and on BMI assessment, this individual will be mistakenly classified as obese.

Thus the identification of individuals with high body fat percentage subclinical is an important measure to identify individuals who need earlier interventions and especially for adolescents. Early detection of excess body fat through low cost and high sensitivity methods allows the implementation of appropriate therapeutic approaches to mitigate the development of comorbidities associated with excess body fat [17]. Therefore, we propose a statistical method to predict obesity in adolescents, using low cost and easily verifiable variables (such as gender, age, height, and body mass).

## 2. Materials and Methods 

### 2.1. Construction of The Database

The database is composed of 722 adolescents. The study included adolescents of both genders, aged 10 to 19 years old, duly enrolled in public schools in São Luís, MA. Exclusion criteria were: pregnancy, lactation, use of birth control pills, being on menstrual period, eating disorders, malnutrition, dehydration, body water retention, refusal to participate in the study, and absenteeism in collections. A single researcher performed each measurement with the same calibrated instrument. Based on the protocol by Lohman [18], the measurements were performed in duplicate, in a single moment (transversal study), and for data analysis, the mean values of the collected measurements were calculated. The variables evaluated were socio-demographic (age and gender) and anthropometric (body mass, height, body circumferences, and body fat percentage). The present study is approved by the Ethics Committee in Research with Human Beings of the Federal University of Maranhão according to legal opinion CAAE: 83206118.1.0000.5087.

### 2.2. Sample Calculation

The sample size was calculated by proportion estimation [19], based on the prevalence of overweight in adolescents of 20.5% [20], which suggested a prevalence of outcome of 26.9% [21], tolerable error of 5% (type I error), and test power of 90% (Type II error), with 10 % added for possible losses or refusals. A minimum sample of 513 teenagers was reached.

### 2.3. Data Collect

Body mass was measured with a calibrated electronic scale (Omron^®^ HBF 214 LA, Japan) with a precision of 0.1 kg. Height was determined through a portable vertical stadiometer with precision of 0.1 cm (Sanny^®^, Brazil). For the measurement of circumferences, an anthropometric tape measure with 0.1 cm precision was used (Seca^®^ 213, Hamburg, Germany). Waist circumference was measured at the midpoint between the last rib and the iliac crest at minimum respiration [22]. Hip circumference was measured at the widest point as described by Huang et al. [23]. Arm circumference was evaluated at midpoint between olecranon and acromial process on the upper left-arm with the subject in standing position [24], and the calf was measured with the participant seated, knee bent at a ninety-degree angle to the floor and this was considered the point of largest calf circumference [25].

The BMI was calculated according to the index of weight (kg)/height^2^ (m), and the cut-off point used for classification of nutritional status was the one recommended by the World Health Organization for gender and age, where >3 percentile and <85 percentile was classified as eutrophic and >85 percentile was overweight [26,27]. The waist-to-height ratio (WHtR) was calculated using the index of waist circumference (cm)/height (cm). For WHtR evaluation, the cutoff point used was 0.5 for both genders and age [28]. Age was calculated as the difference between the date of birth and the date of measurement, and the gender and ethnicity was self-declared by the participant.

The percentage of body fat was obtained through the (calibrated) tetrapolar bioimpedance method (Maltron 906BF^®^, England). All procedures established were followed. It should be noted that the participants were fasting 2 hours before the evaluation, did not drink alcohol or perform vigorous exercises in the 24 hours prior to the exam, and urinated at least 30 minutes before the test. Measurements were taken with the individual in supine position and without any metallic objects on their body [29,30,31]. For the classification of the adolescents’ nutritional status through the body fat percentage, the following cutoff points were used [32,33]: For males, 10.1–20% was considered normal body fat percentage (BFP) and ≥20.1% as high BFP; and for females, 15.1–25% was considered as normal BFP and ≥25.1 as high BFP.

For BFP measurement through DXA, the GE Healthcare Lunar Prodigy device was used, and the scans were analyzed using software version 14.10 (GE Healthcare). This analysis was performed in a subgroup of 12 adolescents also from the public school system of São Luís, MA, in order to show the correlation between the value of BFP obtained by bioimpedance and by DXA. 

### 2.4. Predictor Variables

All the characteristics used as input from the model (Table 1) were chosen based on low cost indicators and easy application. They also had to be described in the literature for the assessment of nutritional status and health of adolescents. The variables—age, body mass, height, and gender—are indexes recommended by the World Health Organization and have been measured in several studies for the nutritional analysis of this population [1,27,28,34,35,36].

Those variables are also used as a criterion for the evaluation and classification of BMI and waist circumference (for example, References [17,22,37]). In contrast to the anthropometric indicators, waist circumference is already a consolidated index for the analysis of central fat and cardiovascular risk [37,38,39]. In addition, the circumference of the arm, hip, and calf are used to assess the nutritional status and population health [22,23,25,40,41,42,43,44,45,46]. Other factors were considered, such as low cost, reproducibility, and accessibility to the entry attributes, especially if the method is used in remote places.

### 2.5. Statistical Model

In many areas of knowledge, such as engineering and health, many problems involve investigating the relationship between two or more variables [47,48,49,50]. Multiple linear regression (MLR) is a statistical technique widely used in the literature to verify the relationship between a dependent variable and several independent variables [51]. Therefore, to build the computational model to predict body fat percentage, the concept of multiple linear regression was applied, and MATLAB^®^ was used to build the model.

The MLR is based on least squares [51], which minimizes the error between the actual results (BFP obtained by the BIA) of the model and the expected results of the training set. The multiple linear regression aims to find an estimate of the real output by means of an equation, according to Equation (1):

(1) $$y=x_{i}\beta_{i}+\ldots +x_{n}\beta_{n}+\varepsilon$$

where y represents the dependent variable, $x_{i}$ the independent variables, $\beta_{i}$ indicates the regression coefficients, and ε is the error term.

Our model has eight predictive (independent) variables described in Table 1, and the model output (dependent variable) will be the estimated body fat percentage value. After obtaining the BFP value through the model, the participant’s nutritional status was assessed using the Lohman study cutoff point [32,33].

For generalization, and in order to avoid overfitting in the proposed method, the K-fold cross validation test was used, which consists of dividing the data into training and testing sets, where the data is equally divided into equal or nearly equal k segments. In these partitioned folds, both the training and the test are performed through k iterations. In each iteration, we leave a fold to test and train the model in the remaining k-1 folds [52]. Based on Lopes et al. [53], Afzal et al. [54], Song et al. [50], and Chang et al. [55], our dataset was randomly divided into five subsets (k = 5). Thus, from the 772 adolescents, 618 were randomly chosen to compose the training set and 154 were used as the test set (i.e., a proportion of 80% of the data for training and 20% for testing).

### 2.6. Performance Analysis

The performance of the method was evaluated in regard to the sensitivity (Sens—percentage of the cases that are correctly identified as true), specificity (Spe—percentage of the cases that are correctly identified as false), and accuracy (Accu—percentage of the cases that are correctly identified among all subjects). To obtain these measurements, the values of true positives (TP), false positives (FP), true negatives (TN), and false negatives (FN) were calculated [56]. The area under receiver operating characteristic (AUROC) and confidence intervals were determined. The cutoff points used for BMI, WHtR, and BFP performance analysis are described in Section 2.3.

### 2.7. Statistical Analysis

For the statistical analysis, the SPSS software (Statistical Package for the Social Sciences, Inc., Chicago, IL, USA) version 25.0 was used. Data was treated with descriptive procedures (median and interquartile range). To compare groups with normal and altered BFP, the Mann–Whitney U test was used. The Chi-squared test was performed to verify the frequency of categorical variables. Pearson’s correlation was used to evaluate the degree of correlation between the estimated value by the model and the real one (obtained by BIA), as well as for the analysis of the correlation between BIA value obtained by the BIA and DXA value. The results were considered statistically significant if the p-value was < 0.05.

## 3. Results

Table 2 presents the general characteristics of the sample composed of 772 adolescents aged 10 to 19 years.

**Figure 1:**
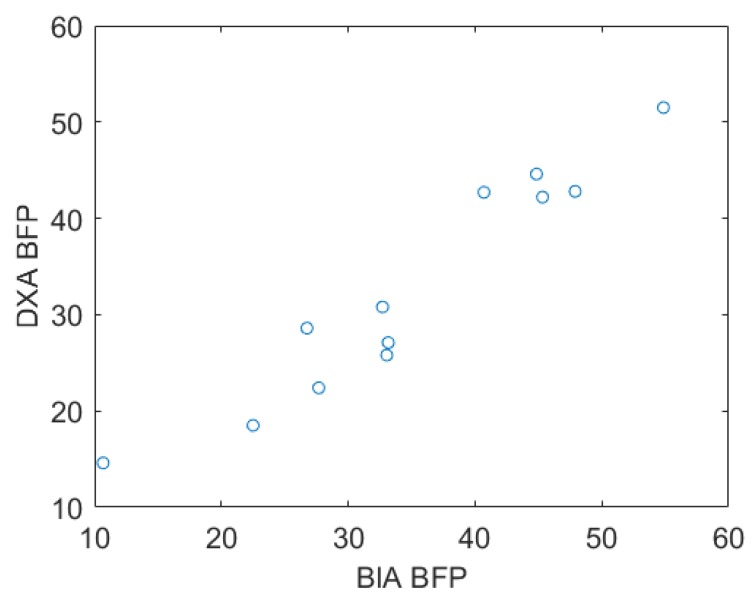
Relation between the DXA BFP and BIA BFP. BFP—body fat percentage; DXA—dual energy X-ray absorptiometry; BIA—Bioimpedance.

The BFP obtained by BIA showed a high correlation (R = 0.96) with DXA BFP in the validation subgroup (n = 12), with a confidence interval of 0.85–0.98. Figure 1 presents the relationship between the DXA value and the BIA BFP value, demonstrating the validity of BIA for BFP estimation in this population.

When comparing the model in relation to the body fat percentage (BIA), there was a significant association between the BFP and the chosen input attributes (Table 3).

**Figure 2:**
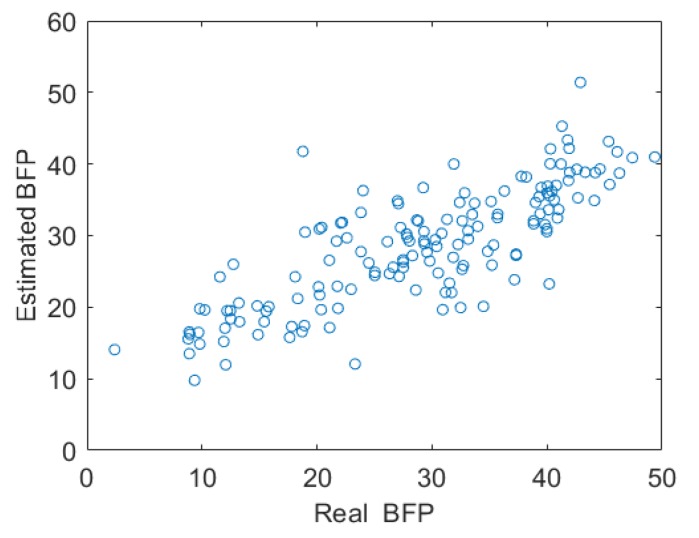
Relation between the real value (BIA) and value estimated by the proposed method. Abbreviations: BFP—body fat percentage.

The proposed method presented high correlation with the BFP value obtained through bioimpedance (R = 0.80), with a confidence interval of 0.73–0.85. Figure 2 presents the relation between the real value (BIA) and the value estimated by our method. 

In Table 4, it is observed that BMI and WHtR present a low AUROC discriminatory power when compared to the proposed method. The WHtR showed low performance compared to the other methods evaluated, presenting a confidence interval less than 0.5 and low sensitivity. Similarly to BMI, the WHtR failed to diagnose more than 50% of the sample of adolescents with excess body fat.

**Figure 3:**
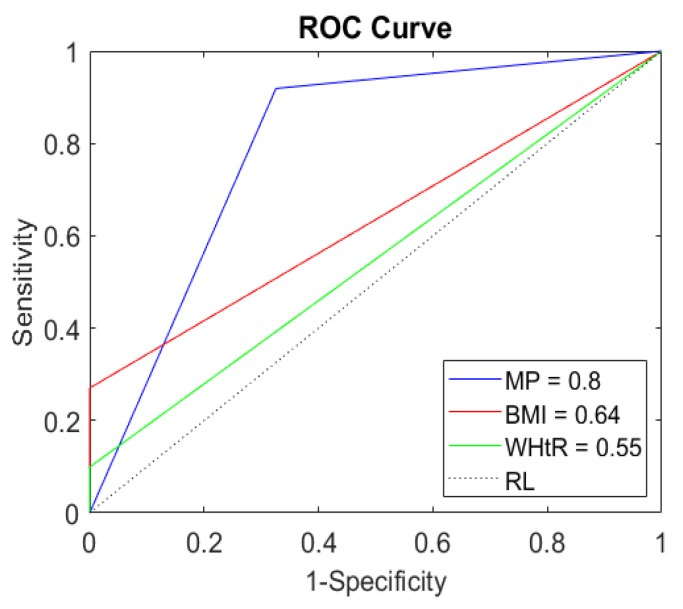
Area under Receiver Operating Characteristic demonstrating the abilities of discrimination of the model and of the anthropometric indicators in the prediction of high body fat in adolescents in the dataset. Abbreviations: MP—proposed method; WHtR—waist-to-height ratio; BMI—body mass index; RL—reference line.

To verify the performance of our method, during the test phase and relative to the obesity indicators commonly used in clinical practices and epidemiological studies, the performance indicators AUROC, precision, sensitivity, and specificity were analyzed. Our model presented excellent discriminatory power, with respect to AUROC, as shown in Table 4 and represented in Figure 3.

Besides, our model showed a better performance than the BMI and WHtR indicators, having high sensitivity with respect to these indicators (Table 4). The development and implementation of a sensible model is of great importance, as a screening method must present high sensitivity, especially if it is used in the analysis of high body fat in adolescents.

Figure 3 is a graphical representation of AUROC, which is generated by plotting a routine (true positive rate) without axis in relation to a specificity (false positive rate) on the x-axis. Thus, for a diagnostic test to be ingested, it is necessary to have a curve without the upper left triangle above the reference line. When higher, it will be better than the next model [57].

Adolescence can be divided into three stages [58]: early adolescence (10–14 years of age), late adolescence (15–19 years of age), and young adults (20–24 years of age). Our test set was divided into two groups, taking into account the gender: 10–14 years of age (precocious adolescence) and 15–19 years of age (late adolescence). For all groups, the measures of accuracy, sensitivity, and specificity were calculated in order to evaluate our method against the anthropometric indicators. The values of accuracy, sensitivity, and specificity are set forth in Table 5. Our method continued to present better sensitivity than the BMI and WHtR anthropometric indices (Table 5).

## 4. Discussion

Adolescence is a stage of physical and intellectual changes [2,59] where there is intense body growth that interferes with the accumulation and distribution of body fat [44]. It is therefore seen as one of the most critical periods for the development of obesity [17]. In the assessment of nutritional status, one of the most relevant indicators is the percentage of body fat (PBF), which is an independent risk factor for insulin resistance and a strong predictor of morbidity, as well as being a key parameter for the preventive and therapeutic intervention of pediatric obesity [60]. 

Measurements of body composition derived from BIA are valuable for the analysis of the nutritional status of pediatric patients [11,61]. It is one of the most reliable and affordable methods for assessing body fat and has a high correlation (r = 0.96–0.92) with dual energy X-ray absorptiometry [9], corroborating our results (r = 0.96). Despite the advantages, BIA still has a high cost when compared to the use of anthropometric indicators and clinical variables.

Thus, we built a method to assess nutritional status in adolescents by estimating body fat percentage using low cost and easy application parameters. The method used for estimation was the MLR, which was already successfully applied to solve several problems such as clinical data analysis [47] to verify the association between autonomic cardiac function and clinical variables [49]; to investigate the effects of food contamination on gastrointestinal tract morbidity [50]; and soil density measurement [48].

In the assessment of excess body fat, the proposed method obtained better performance (accuracy, sensitivity, and AUROC) than the anthropometric indicators BMI and WHtR, which are usually used to assess the nutritional status of adolescents. It is an attractive method with low cost and easy application when compared to other methods of body composition analysis such as bioimpedance. The best performance of our method can be explained by the use of predictor variables, such as body circumferences (Table 1), which are widespread measures in the literature for the assessment of obesity in this population and are associated with the presence of visceral fat and cardiovascular risk factors [38,62,63,64], as well as the use of indicators such as gender, age, height, and body mass together, which are widely used in the analysis of the nutritional profile and health status of the juvenile population [1,17,34,65]. 

The body mass index, despite being the most used method in clinical practice and epidemiological studies to assess excess body fat [21,66], is widely criticized for not correlating with body composition and being a poor predictor of body fat [9,12,15,60,67]. In a recent review, it was observed that BMI has high specificity (approximately 92%) and low sensitivity (approximately 50 %) to detect obesity based on body fat percentage [68,69]. Therefore, more than half of individuals could be mistakenly classified with normal BFP by BMI [68], corroborating the results of the present study.

This fact is concerning because the low sensitivity of BMI indicates that excess adiposity is being underdiagnosed in several individuals. Moreover, because the first step in dealing with a risk factor is the precise identification of the pathophysiological problem, late diagnosis of excess adiposity will delay the treatment of associated comorbidities, as well as the implementation of intervention and control measures [68,70,71], especially in the juvenile population [71].

Although used in several studies with adolescents, WHtR did not show efficient discriminatory power in the evaluation of adolescents with high body fat in the present study. In a systematic review, Lo et al. [72] observed that WHtR did not perform better in predicting cardiometabolic risk factors than anthropometric indicators BMI and WC (waist circumference) in children and adolescents.

Thus, the use of high sensitivity methods is a major challenge for the early diagnosis of obese adolescents. The model proposed here has predictive and practical advantages in situations with limited resources, such as areas without access to equipment for body composition analysis (bioimpedance, for example). Thus, this is an alternative for screening adolescents for excess body fat. In addition, such a method may guide health professionals in decision-making and potentially expedite tests such as lipid profile and insulin resistance, which are associated with high body fat levels and cardiovascular risk.

A limitation of this study was to use as a test set approximately 20 % of the sample, since a larger sample could provide more robust results. However, in this study, the main goal was to present a new method for screening obesity in adolescents. The authors believe that an external validation study should be performed in other regions of Brazil and other countries due to variations in ethnic descent.

## 5. Conclusions

The computational model of this study obtained a better performance in the evaluation of excess body fat in adolescents compared to the usual anthropometric indicators, thus presenting itself as a low cost alternative for screening obesity in adolescents living in Brazilian regions where financial resources are scarce.

## Figures and Tables

**Table 1:** Entry attributes in our model.

Attribute	Abbreviation	Unit
Body Mass	BM	Kg
Height	Ht	M
Gender	-	-
Age	-	years
Arm Circumference	AC	cm
Waist Circumference	WC	cm
Calf Circumference	CC	cm
Hip Circumference	HC	cm

Abbreviations: kg—kilogram; m—meters; cm—centimeter.

**Table 2:** Socio-demographic, anthropometric, and hemodynamic characteristics from the database, stratified through body fat percentage (BFP).

Variables	Normal BFP * (n = 233)	Elevated BFP * (n = 539)	All ^#^ (n = 772)
**Ht** (m)	1.67 (1.59−1.75)	1.62 (1.57−1.68)	1.64±0.09
**BM** (kg)	53.6 (45.7−61.5)	56.9(50.1−64.3)	57.18±11.67
**Age** (years)	17 (15−17)	16(15−17)	15.66±1.72
**HC** (cm)	86.5 (82−91)	92 (86.5−97)	90.31±8.6
**WC** (cm)	66 (62−71)	69 (65−75)	69±8.4
**AC** (cm)	23 (21−25.5)	25 (23−28)	24.9±3.90
**CC** (cm)	32 (30−34.7)	34 (31.5−36)	33.2±3.9
**BFP** (%)	15.7(12.7−19.8)	33.7 (28.4−39.1)	28.4±10.23
**Gender** ^§^			
*Female*	99	402	501
*Male*	134	137	271
**Ethnicity** ^§^			
*Caucasian*	53	124	177
*Non-Caucasian*	180	415	595

Abbreviations: Ht—height; BM—body mass; HC—hip circumference; WC—waist circumference; AC—arm circumference; CC—calf circumference; kg—kilogram; m—meters; cm—centimeter; %—percentage; BPF—body fat percentage; * Values are presented as median (interquartile range: 25–75%); # Results are presented using mean ± standard deviation; § Values shown in frequency.

**Table 3:** Regression coefficient (Beta) and significance (*p*) for each analyzed variable of the model, in relation to the body fat percentage.

Attribute	Beta	*p*
Body Mass	−0.12	0.018
Height	−6.84	0.005
Gender	−11.5	<0.001
Age	−1.32	<0.001
Arm Circumference	0.37	0.002
Waist Circumference	0.31	<0.001
Calf Circumference	0.40	0.001
Hip Circumference	0.30	<0.001

**Table 4:** Analysis of the performance in the test set of the proposed methods relative to the anthropometric indicators BMI and WHtR.

Indicators	AUROC * (CI 95 %)	Accu (%)	Sens (%)	Spe (%)	TP (%)	TN (%)	FP (%)	FN (%)
MP	0.80 (0.70–0.90)	85.1	92	67.4	66	19	9	6
BMI	0.64 (0.51–0.77)	47.4	27	100	19	28	0	53
WHtR	0.55 (0.36–0.74)	35.1	9.9	100	7	28	0	65

Abbreviations: CI 95 %—confidence interval; * Area under the ROC curve demonstrating discriminatory power for body fat (lower limit of CI 95 % > 0.50); MP—proposed method; WHtR—waist-to-height ratio; BMI—body mass index; Accu—accuracy; Sens—sensitivity; Spe—specificity; TP—true positives; FP—false positives; TN—true negatives; FN—false negatives; %—percentage.

**Table 5:** Analysis of the performance of the proposed methods in the data set relative to the anthropometric indicators BMI and WHtR, stratified by gender and age.

(%)	Male						Female					
	10–14 (n = 13)			15–19 (n = 44)			10–14 (n = 17)			15–19 (n = 80)		
	MP	BMI	WHtR	MP	BMI	WHtR	MP	BMI	WHtR	MP	BMI	WHtR
Accu	84	46	46	82	73	64	76	53	23	89	32	20
Sens	75	12	12	80	40	20	100	38	0	96	23	8
Spe	100	100	100	83	100	100	0	100	100	40	100	100
TP	46	8	8	36	18	9	76	29	0	84	20	7
TN	39	39	39	46	55	55	0	24	24	5	13	13
FP	0	0	0	9	0	0	24	0	0	7	0	0
FN	15	53	53	9	27	36	0	47	76	4	67	80

Abbreviations: MP—proposed method; WHtR—waist-to-height ratio; BMI—body mass index; Accu—accuracy; Sens—sensitivity; Spe—specificity; TP—true positives; FP—false positives; TN—true negatives; FN—false negatives; %—percentage.

## References

[1] Katia V.C., Moyses S., Maria C.C.K., Gabriela A.A., Laura A.B., Carlos H.K., Maurício T.L.V., Glória V.V., Valeska C.F., Adriano D. (2015). The study of cardiovascular risk in adolescents—ERICA: Rationale, design and sample characteristics of a national survey examining cardiovascular risk factor profile in Brazilian adolescents. BMC Public Health.

[2] Dahl R.E., Allen N.B., Wilbrecht L., Suleiman A.B. (2018). Importance of investing in adolescence from a developmental science perspective. Nature.

[3] Hagani N., Moran M.R., Caspi O., Plaut P., Endevelt R. (2019). The Relationships between Adolescents’ Obesity and the Built Environment: Are They City Dependent?. Int. J. Environ. Res. Public Health.

[4] Reinehr T. (2018). Long-term effects of adolescent obesity: Time to act. Nat. Rev. Endocrinol..

[5] Andreoli A., Garaci F., Cafarelli F.P., Guglielmi G. (2016). Body composition in clinical practice. Eur. J. Radiol..

[6] Tewari N., Awad S., Macdonald I.A., Lobo D.N. (2018). A comparison of three methods to assess body composition. Nutrition.

[7] Böhm A., Heitmann B.L. (2013). The use of bioelectrical impedance analysis for body composition in epidemiological studies. Eur. J. Clin. Nutr..

[8] Dehghan M., Merchant A.T. (2018). Is bioelectrical impedance accurate for use in large epidemiological studies?. Nutr. J..

[9] Ramírez-Vélez R., Correa-Bautista J., Martínez-Torres J., González-Ruíz K., González-Jiménez E., Valle J., Ramírez-Vélez R. (2016). Performance of Two Bioelectrical Impedance Analyses in the Diagnosis of Overweight and Obesity in Children and Adolescents: The FUPRECOL Study. Nutrients.

[10] Tovar-Galvez M.I., González-Jiménez E., Martí-García C., Schmidt-RioValle J. (2017). Composición corporal en escolares: Comparación entre métodos antropométricos simples e impedancia bioeléctrica. Endocrinol. Diabetes Nutr..

[11] Von Hurst P.R., Walsh D.C.I., Conlon C.A., Ingram M., Kruger R., Stonehouse W. (2016). Validity and reliability of bioelectrical impedance analysis to estimate body fat percentage against air displacement plethysmography and dual-energy X-ray absorptiometry. Nutr. Diet..

[12] Guo B., Wu Q., Gong J., Xiao Z., Tang Y., Shang J. (2015). Relationships between the lean mass index and bone mass and reference values of muscular status in healthy Chinese children and adolescents. J. Bone Miner. Metab..

[13] Laurson K.R., Welk G.J., Eisenmann J.C. (2014). Diagnostic Performance of BMI Percentiles to Identify Adolescents With Metabolic Syndrome. Pediatrics.

[14] de Oliveira P.M., Da Silva F.A., Oliveira R.M.S. (2016). Association between fat mass index and fat-free mass index values and cardiovascular risk in adolescents. Revista Paulista de Pediatria (English Edition).

[15] de Cássia Ribeiro-Silva R., Florence T., da Conceição-Machado M.E.P., Fernandes G.B., Couto R.D. (2014). Indicadores antropométricos na predição de síndrome metabólica em crianças e adolescentes: um estudo de base populacional. Rev. Bras. Saúde Matern. Infant..

[16] Weber D.R., Leonard M.B., Zemel B.S. (2012). Body composition analysis in the pediatric population. Pediatr. Endocrinol. Rev..

[17] ANS (2017). Agência Nacional De Saúde Suplementar Diretoria De Normas E Habilitação Dos Produtos Gerência De Monitoramento Assistencial Coordenadoria De Informações Assistenciais. http://www.ans.gov.br/images/Manual_de_Diretrizes_para_o_Enfrentamento_da_Obesidade_na_Saúde_Suplementar_Brasileira.pdf.

[18] Lohman T.G. (1986). Applicability of body composition techniques and constants for children and youths. Exerc. Sport Sci. Rev..

[19] Lwanga S., Lemeshow S. (1991). Sample Size Determination in Health Studies: A Practical Manual.

[20] Oliveira MLDE (2013). Estimativa Dos Custos Da Obesidade Para O Sistema Único de Saúde do Brasil. http://repositorio.unb.br/handle/10482/13323.

[21] Farias E.D.S. (2012). Excess weight and associated factors in adolescents. Rev. Nutr..

[22] Brasil A (2016). Associação Brasileira para o Estudo da Obesidade e da Síndrome Metabólica. http://www.abeso.org.br/uploads/downloads/92/57fccc403e5da.pdf.

[23] Huang B.X., Zhu M.F., Wu T., Zhou J.Y., Liu Y., Chen X.L., Zhou R.F., Wang L.J., Chen Y.M., Zhu H.L. (2015). Neck circumference, along with other anthropometric indices, has an independent and additional contribution in predicting fatty liver disease. PLoS ONE.

[24] Rerksuppaphol S., Rerksuppaphol L. (2017). Mid-upper-arm circumference and arm-to-height ratio to identify obesity in school-age children. Clin. Med. Res..

[25] Salmaso F.V., Vigário P.D.S., Mendonça L.M.C.D., Madeira M., Netto L.V., Guimarães M.R.M., Farias M.L.F.D. (2014). Análise de idosos ambulatoriais quanto ao estado nutricional, sarcopenia, função renal e densidade óssea. Arq. Bras. Endocrinol. Metab..

[26] Brasil. Ministério da Saude (2011). Orientações para a Coleta e Análise de Dados Antropométricos em Serviços de Saúde: Norma Técnica do Sistema de Vigilância Alimentar e Nutricional - SISVAN.

[27] Onis M.D. (2007). WHO Child Growth Standards based on length/height, weight and age. Acta Pædiatr..

[28] Ashwell M., Hsieh S.D. (2005). Six reasons why the waist-to-height ratio is a rapid and effective global indicator for health risks of obesity and how its use could simplify the international public health message on obesity. Int. J. Food Sci. Nutr..

[29] Houtkooper L.B., Going S.B., Lohman T.G., Roche A.F., Van Loan M. (1992). Bioelectrical impedance estimation of fat-free body mass in children and youth: A cross-validation study. J. Appl. Physiol..

[30] Kyle U.G., Bosaeus I., De Lorenzo A.D., Deurenberg P., Elia M., Gómez J.M. (2004). Bioelectrical impedance analysis—Part I: Review of principles and methods. Clin. Nutr..

[31] Neves E.B., Ripka W.L., Ulbricht L., Stadnik A.M.W. (2013). Comparison of the fat percentage obtained by bioimpedance, ultrasound and skinfolds in young adults. Rev. Bras. Med. Esporte.

[32] Lohman T.G. (1993). Advances in body composition assessment. Med. Sci. Sport Exerc..

[33] Ripka W.L. (2017). Modelos Matemáticos Para Estimativa da Gordura Corporal de Adolescentes Utilizando Dobras Cutâneas, a Partir da Absorciometria de Raios-X de Dupla Energia. Ph.D. Thesis.

[34] Jésus P., Guerchet M., Pilleron S., Fayemendy P., Maxime Mouanga A., Mbelesso P. (2017). Undernutrition and obesity among elderly people living in two cities of developing countries: Prevalence and associated factors in the EDAC study. Clin. Nutr. ESPEN.

[35] Sigulem D.M., Devincenzi M.U., Lessa A.C. (2000). Diagnosis of the nutritional status of children and adolescents. J. Pediatr..

[36] Onis M.D., Onyango A.W., Borghi E., Siyam A., Nishida C., Siekmann J. (2007). Development of a WHO growth reference for school-aged children and adolescents. Bull. World Health Organ..

[37] Taylor R.W., Jones I.E., Williams S.M., Goulding A. (2000). Evaluation of waist circumference, waist-to-hip ratio, and the conicity index as screening tools for high trunk fat mass, as measured by dual-energy X-ray absorptiometry, in children aged 3-19 y13. Am. J. Clin. Nutr..

[38] Savva S.C., Tornaritis M., Savva M.E., Kourides Y., Panagi A., Silikiotou N. (2000). Waist circumference and waist-to-height ratio are better predictors of cardiovascular disease risk factors in children than body mass index. Int. J. Obes..

[39] Schröder H., Ribas L., Koebnick C., Funtikova A., Gomez S.F., Fíto M. (2014). Prevalence of abdominal obesity in Spanish children and adolescents. do we need waist circumference measurements in pediatric practice?. PLoS ONE.

[40] Alencar M.D.S.S., Melo M.T.S.M., Coê R., Meneses A.V., Sá L.D., Nunes I.F.D.O.C. (2015). Loss of muscle and fat mass after institutionalization: Attention to older. Geriatr. Gerontol. Aging.

[41] de Faria E.R., Gontijo C.A., Franceschini S.D.C.C., Peluzio M.D.C.G., Priore S.E. (2014). Body composition and risk for metabolic alterations in female adolescents. Rev. Paul. Pediatr..

[42] Kroll C., Mastroeni S.S.B.S., Czarnobay S.A., Ekwaru J.P., Veugelers P.J., Mastroeni M.F. (2017). The accuracy of neck circumference for assessing overweight and obesity: a systematic review and meta-analysis. Ann. Hum. Biol..

[43] Li C., Ford E.S., Mokdad A.H., Cook S. (2006). Recent Trends in Waist Circumference and Waist-Height Ratio Among US Children and Adolescents. Pediatrics.

[44] Pereira P.F., de Faria F.R., de Faria E.R., Hermsdorff H.H.M., Peluzio M.D.C.G., Franceschini S.D.C.C., Priore S.E. (2015). Anthropometric indices to identify metabolic syndrome and hypertriglyceridemic waist phenotype: a comparison between the three stages of adolescence. Rev. Paul. Pediatr..

[45] Ripka W.L., Ulbricht L., Gewehr P.M. (2017). Body composition and prediction equations using skinfold thickness for body fat percentage in Southern Brazilian adolescents. PLoS ONE.

[46] Talma H., Van Dommelen P., Schweizer J.J., Bakker B., Kist-Van Holthe J.E., Chinapaw J.M.M. (2019). Is mid-upper arm circumference in Dutch children useful in identifying obesity?. Arch. Dis. Child..

[47] Coelho-Barros E.A., Simões P.A., Achcar J.A., Martinez E.Z., Shimano A.C. (2008). Methods of Estimation in Multiple Linear Regression: Application to Clinical Data. Rev. Colomb. Estad..

[48] Carvalho Junior W.D., Filho B.C., Chagas C.D.S., Bhering S.B., Pereira N.R., Pinheiro H.S.K. (2016). Multiple linear regression and Random Forest model to estimate soil bulk density in mountainous regions. Pesqui. Agropecu. Bras..

[49] Santos R.R., Rosa E.C., Rosa T., Ferreira E.A., Gris E.F., de Andrade R.V. (2019). Sedentary Behavior: A Key Component in the Interaction between an Integrated Lifestyle Approach and Cardiac Autonomic Function in Active Young Men. Int. J. Environ. Res. Public Health.

[50] Song Q., Zheng Y.J., Yang J. (2019). Effects of Food Contamination on Gastrointestinal Morbidity: Comparison of Different Machine-Learning Methods. Int. J. Environ. Res. Public Health.

[51] Akan R., Keskin S.N., Uzundurukan S. (2015). Multiple Regression Model for the Prediction of Unconfined Compressive Strength of Jet Grout Columns. Procedia Earth Planet. Sci..

[52] Yadav S., Shukla S. Analysis of k-Fold Cross-Validation over Hold-Out Validation on Colossal Datasets for Quality Classification. Proceedings of the 6th International Advanced Computing Conference, IACC 2016.

[53] Lopes M.V., Barradas Filho A.O., Barros A.K., Viegas I.M.A., Silva L.C.O., Marques E.P. (2019). Attesting compliance of biodiesel quality using composition data and classification methods. Neural Comput. Appl..

[54] Afzal Z., Schuemie M.J., Van Blijderveen J.C., Sen E.F., Sturkenboom M.C., Kors J.A. (2013). Improving sensitivity of machine learning methods for automated case identification from free-text electronic medical records. BMC Med. Inf. Decis. Mak..

[55] Chang F., Chen H.C., Liu H.C. Double K-Folds in SVM. Proceedings of the 9th International Conference on Innovative Mobile and Internet Services in Ubiquitous Computing, IMIS 2015.

[56] Rovini E., Maremmani C., Moschetti A., Esposito D., Cavallo F. (2018). Comparative Motor Pre-clinical Assessment in Parkinson’s Disease Using Supervised Machine Learning Approaches. Ann. Biomed. Eng..

[57] Hoo Z.H., Candlish J., Teare D. (2017). What is an ROC curve?. Emerg. Med. J..

[58] Das J.K., Salam R.A., Thornburg K.L., Prentice A.M., Campisi S., Lassi Z.S. (2017). Nutrition in adolescents: physiology, metabolism, and nutritional needs. Ann. NY Acad. Sci..

[59] Moraes M.M.D., Veiga G.V.D. (2014). Acurácia da gordura corporal e do perímetro da cintura para predizer alterações metabólicas de risco cardiovascular em adolescentes. Arq. Bras. Endocrinol. Metabol..

[60] Vanderwall C., Randall Clark R., Eickhoff J., Carrel A.L. (2017). BMI is a poor predictor of adiposity in young overweight and obese children. BMC Pediatr..

[61] Kyle U.G., Earthman C.P., Pichard C., Coss-Bu J.A. (2015). Body composition during growth in children: limitations and perspectives of bioelectrical impedance analysis. Eur. J. Clin. Nutr..

[62] Brambilla P., Bedogni G., Moreno L.A., Goran M.I., Gutin B., Fox K.R. (2006). Crossvalidation of anthropometry against magnetic resonance imaging for the assessment of visceral and subcutaneous adipose tissue in children. Int. J. Obes..

[63] Rerksuppaphol S., Rerksuppaphol L. (2014). Waist circumference, waist-to-height ratio and body mass index of thai children: Secular changes and updated reference standards. J. Clin. Diagn. Res..

[64] Tebar W.R., Ritti-Dias R.M., Farah B.Q., Zanuto E.F., Vanderlei L.C.M., Christofaro D.G.D. (2018). High blood pressure and its relationship to adiposity in a school-aged population: Body mass index vs waist circumference. Hypertens. Res..

[65] Sousa N.P.S., Salvador E.P., Barros A.K., Polisel C.G., de Carvalho W.R.G. (2016). Anthropometric predictors of abdominal adiposity in adolescents. J. Exerc. Physiol. Online.

[66] Lavrador M.S.F., Abbes P.T., Escrivão M.A.M.S., Taddei J.A.A.C. (2011). Riscos cardiovasculares em adolescentes com diferentes graus de obesidade. Arquivos Brasileiros de Cardiologia.

[67] De Lorenzo A., Soldati L., Sarlo F., Calvani M., Di Lorenzo N., Di Renzo L. (2016). New obesity classification criteria as a tool for bariatric surgery indication. World J. Gastroenterol..

[68] Oliveros E., Somers V.K., Sochor O., Goel K., Lopez-Jimenez F. (2014). The Concept of Normal Weight Obesity. Prog. Cardiovasc Dis..

[69] Frayon S., Cavaloc Y., Wattelez G., Cherrier S., Lerrant Y., Ashwell M. (2018). Potential for waist-to-height ratio to detect overfat adolescents from a Pacific Island, even those within the normal BMI range. Obes. Res. Clin. Pract..

[70] Javed A., Jumean M., Murad M.H., Okorodudu D., Kumar S., Somers V.K. (2015). Diagnostic performance of body mass index to identify obesity as defined by body adiposity in children and adolescents: A systematic review and meta-analysis. Pediatr. Obes..

[71] Zhang S., Tjortjis C., Zeng X., Qiao H., Buchan I., Keane J. (2009). Comparing data mining methods with logistic regression in childhood obesity prediction. Inf. Syst. Front..

[72] Lo K., Wong M., Khalechelvam P., Tam W. (2016). Waist-to-height ratio, body mass index and waist circumference for screening paediatric cardio-metabolic risk factors: A meta-analysis. Obes. Rev..

